# Circulating Levels of Human salusin-β,a Potent Hemodynamic and Atherogenesis Regulator

**DOI:** 10.1371/journal.pone.0076714

**Published:** 2013-10-03

**Authors:** Kazumi Fujimoto, Akinori Hayashi, Yuji Kamata, Akifumi Ogawa, Takuya Watanabe, Raishi Ichikawa, Yoshitaka Iso, Shinji Koba, Youichi Kobayashi, Takatoshi Koyama, Masayoshi Shichiri

**Affiliations:** 1 Laboratory of Molecular Genetics of Hematology, Graduate School of Health Care Sciences, Tokyo Medical and Dental University, Tokyo, Japan; 2 Department of Endocrinology, Diabetes and Metabolism, Kitasato University School of Medicine, Kanagawa, Japan; 3 Laboratory of Cardiovascular Medicine, Tokyo University of Pharmacy and Life Sciences, Tokyo, Japan; 4 Division of Cardiology, Department of Medicine, Showa University School of Medicine, Tokyo, Japan; University Medical Center Utrecht, Netherlands

## Abstract

Using bioinformatics analysis, we previously identified salusin-β, an endogenous bioactive peptide with diverse physiological activities. Salusin-β is abundantly expressed in the neuroendocrine system and in systemic endocrine cells/macrophages. Salusin-β acutely regulates hemodynamics and chronically induces atherosclerosis, but its unique physicochemical characteristics to tightly adhere to all types of plastic and glassware have prevented elucidation of its precise pathophysiological role. To quantitate plasma total salusin-β concentrations, we produced rabbit and chicken polyclonal antibodies against the C- and N-terminal end sequences, circumvented its sticky nature, and successfully established a sandwich enzyme-linked immunosorbent assay (ELISA). Salusin-β was abundantly present in the plasma of healthy volunteers, ranging from 1.9 to 6.6 nmol/L. Reverse phase-high performance liquid chromatography analysis showed that a single immunoreactive salusin-β peak coincided with synthetic authentic salusin-β. Plasma salusin-β concentrations were unaffected by postural changes and by potent vasopressin release stimuli, such as hypertonic saline infusion or smoking. However, salusin-β concentrations showed significant circadian variation; concentrations were high during the daytime and reached the lowest concentrations in the early morning. Plasma salusin-β levels in subjects with diabetes mellitus, coronary artery disease, and cerebrovascular disease showed distinctly higher levels than healthy controls. Patients with panhypopituitarism combined with complete central diabetes insipidus also showed significantly higher plasma salusin-β levels. Therefore, the ELISA system developed in this study will be useful for evaluating circulating total salusin-β levels and for confirming the presence of authentic salusin-β in human plasma. The obtained results suggest a limited contribution of the neuroendocrine system to peripheral total salusin-β concentrations and a role for plasma total salusin-β concentrations as an indicator of systemic vascular diseases.

## Introduction

Immunoreactive salusin-β is localized to the neuroendocrine system in the brain and is also present throughout systemic endocrine cells and certain hematopoietic cells, such as macrophages [1-4]. Salusin-β stimulates the release of vasopressin and oxytocin from the posterior pituitary [3,5], and induces rapid and profound decreases in blood pressure and heart rate [5,6], while endogenous salusin-β in the vasculature may act to promote atherosclerosis [5-8]. Despite such unique and diverse physiological actions, elucidation of salusin-β’s pathophysiological roles has been precluded by its peculiar physicochemical features to adhere to all sorts of plastic and glass items for medical and laboratory use [9,10]. Many attempts to establish an accurate bioassay system have been unsuccessful, leaving the clinical application of salusin-β unavailable. By using a low dose of non-ionic detergents to circumvent these properties, we previously established a radioimmunoassay and demonstrated the presence of salusin-β-like immunoreactivity in normal human plasma and urine [11]. However, low antigenicity of N-terminal amino acid residues of salusin-β has prevented achievement of a highly sensitive bioassay to determine its plasma levels. In this study, we used a novel strategy to produce polyclonal antiserum against such an amino acid sequence of low antigenicity [12] and successfully established a sandwich enzyme-linked immunosorbent assay (ELISA) suitable for detection of salusin-β in human plasma. This allowed us to investigate the physiological and pathophysiological significance of circulating salusin-β in humans.

## Patients and Methods

### Subjects

The study population consisted of 106 healthy volunteers (64 men and 42 women, aged between 21 and 59 years) and 113 patients (69 men and 44 women, aged between 20 and 95 years) with the definite diagnosis of the following diseases: coronary artery disease (30 men and 7 women), cerebrovascular disease (17 men and 26 women), diabetes (19 men and 9 women), and panhypopituitarism with complete central diabetes insipidus (3 men and 2 women). None of the healthy volunteers had any current medical problems and were receiving any medications. Coronary artery disease was defined by the presence of ≥ 75% diameter stenosis on coronary angiography and cerebrovascular disease on a brain computed tomography scan and/or magnetic resonance imaging. Diabetes was defined by Japan Diabetes Society criteria. Patients with panhypopituitarism/diabetes insipidus had clear-cut evidence of a deficient posterior pituitary caused either by a pituitary tumor or complete severance of the pituitary stalk, and received replacement therapy consisting of levothyroxine, cortisol and desmopressin. This study was approved by the Ethics Committees of Kitasato University Hospital, Tokyo Medical and Dental University, Tokyo University of Pharmacy and Life Sciences, and Showa University. All participants provided written or witnessed verbal informed consent. A verbal informed consent was witnessed by a family member and documented in individual medical records. This process was allowed in the Ethics Committees of Tokyo University of Pharmacy and Life Sciences and Showa University in order to not exclude acute vascular disease participants. No minors/children were involved in the study, so no informed consent was obtained from next of kin, caretakers or guardians.

### Sample collection and extraction of plasma

Baseline blood samples were collected from the above subjects and patients into vacutainers containing Na_2_-EDTA (1.5 mg/mL) or sodium citrate (3.13%), and plasma was separated immediately in a refrigerated centrifuge and stored in aliquots at -30°C until processing. The plasma was extracted essentially as previously described [11] but with the following modification. Thawed plasma was acidified with 0.1% of trifluoroacetic acid (TFA), centrifuged at 1500 × *g* for 10 min, and applied to a prewashed Sep-Pak C18 cartridge (Waters Associates, Milford, MA, USA). The materials adsorbed onto the cartridge were eluted with 1 ml of 70% acetonitrile in 0.1% TFA into polypropylene tubes already containing 10 µl of 10% NP-40. They were immediately mixed, and evaporated to approximately 100 µl using a centrifugal concentrator (2000 rpm).

### Antibodies

Polyclonal antiserum against C-terminal salusin-β was raised in two Japanese white rabbits by immunization with synthetic [Cys^0^]-salusin-β coupled to maleimide-activated mariculture keyhole limpet hemocyanin (Pierce, Rockford, IL, USA). Polyclonal antiserum against N-terminal salusin-β was produced in chicken by immunization with a synthetic N-terminal salusin-β sequence, AIFIFI, pretreated with a protein crosslinking and fixation reagent, as previously described [12]. Chicken IgY was extracted from egg yolk using the Thermo Scientific Pierce Chicken IgY Purification Kit (Thermo, Fisher Scientific, Waltham, MA, USA) according to the manufacturer’s instructions.

### Salusin-β ELISA

A sandwich ELISA was established to measure immunoreactive salusin-β in extracted plasma. All washes were performed three times with phosphate-buffered saline (PBS) (pH 7.4) containing 0.05% Tween 20. Flat-bottom 96-well plates (Asahi Glass, Funabashi, Japan) were coated with 100 µl of anti-N-terminal salusin-β IgY antibody (1: 80000 dilution) in PBS and incubated overnight at 4°C. The plates were washed and blocked with 100 µl of 10% w/v skim milk in Tris-buffered saline for 2 h at room temperature. After washing, 50 µl of the standard salusin-β (from 0.2 nmol/l to 2.0 nmol/l) or extracted human plasma was added in triplicate and incubated for 1 h at room temperature. Three wells did not receive any standard peptide or samples at this step, but the other ELISA steps were performed as those for other wells. The plates were washed again, incubated for 1 h with anti-C-terminal salusin-β antibody (1:50000 dilution), and further incubated for 1 h with horseradish peroxidase-conjugated donkey anti-rabbit IgG (GE Healthcare, Amersham, Buckinghamshire, UK) (1:2000 dilution). After washing, a solution containing 0.04% H_2_O_2_ and orthophenylenediamine (Sigma Aldrich, St. Louis, MO, USA) dissolved in citrate-sodium phosphate buffer was added to each well. The reaction was stopped with 0.5 M H_2_SO_4_ after 30 min, and the absorbance was measured at 490 nm with a microplate reader (Bio-Rad, Tokyo, Japan). Blank wells received only 0.1% NP40/PBS before measuring absorbance.

### Reverse-phase high performance liquid chromatography (RP-HPLC)

Extracted plasma samples were loaded onto a μRPCC2/C18 ST 4.6/100 reverse-phase column (GE Healthcare, UK) and separated using a linear gradient of acetonitrile from 0 to 100% in 0.1% TFA as previously described [13,14]. The column was run at a flow rate of 0.5 ml/min for 80 min using an AKTA Explorer 10S HPLC system (GE Healthcare). A total of 1.0 ml fractions were collected in polypropylene tubes already containing 10 µl of 10% NP-40, evaporated, reconstituted, and subjected to the ELISA. Synthetic salusin-β (Peptide Institute, Osaka, Japan) was reconstituted in 0.1% NP-40 and subjected to RP-HPLC analysis.

### Clinical protocols

#### Postural changes

Seven healthy subjects (4 men and 3 women) fasted overnight and first sat on a chair for 15 min. After this time, they lay supine for 30 min, and then for a final 60-min period they either kept standing or walking without sitting. A blood sample was taken from an arm vein at the end of sitting, supine, and standing periods.

#### Circadian variation

All 30 healthy subjects (20 men and 10 women) received blood collection during the daytime either at our clinical research ward or medical school laboratory. Throughout the study period, usual ambulant activity was allowed. Blood samples were collected at 9:00, 10:00, 12:00, 14:00, 16:00, and 18:00 h. Eight of these volunteers participated in overnight blood sampling through an intravenous catheter inserted into a suitable antecubital vein and received additional blood collections at 20:00, 23:00 3:00, 6:00, 7:00 and 8:00 h. They were assumed to sleep from 11 pm to 6 am and got up at 6 am.

#### Oral water loading

The response to oral water load was studied in nine healthy volunteers (6 men and 3 women). Following an overnight fast, a blood sample was withdrawn at 10 am and the subjects were given an oral water load of 20 ml/kg body weight within 20 min. Subjects remained in the sitting position without voiding until blood was again collected 1 h later.

#### Intravenous hypertonic saline infusion

Six healthy subjects (4 men and 2 women) were given hypertonic saline intravenously at a recumbent position in the morning following an overnight fast. After 30 min recumbency, intravenous access was obtained around an antecubital fossae and baseline blood samples were obtained. An infusion of 5% of NaCl then took place at a constant rate of 0.05 ml/kg/min over 2 h. Blood was drawn at 30, 60, 90, and 120 min after the start of the infusion for the measurement of salusin-β.

#### Smoking

Six current male smokers refrained from drinking alcohol and smoking from the night before the test. Baseline blood samples were collected before 9:30 am, and at 10 am, the participants smoked one filtered cigarette containing 0.6 mg of nicotine within 4 min. Blood samples were then collected 15, 30, and 60 min after the initiation of smoking.

### Statistical analysis

Data are expressed as the mean ± SD. Differences between groups were examined for statistical significance using the Mann-Whitney test or paired Wilcoxon signed-rank test using GraphPad Prism 5 (GraphPad Software, La Jolla, CA, USA). *P* ˂ 0.05 was considered statistically significant.

## Results

**Figure 1:**
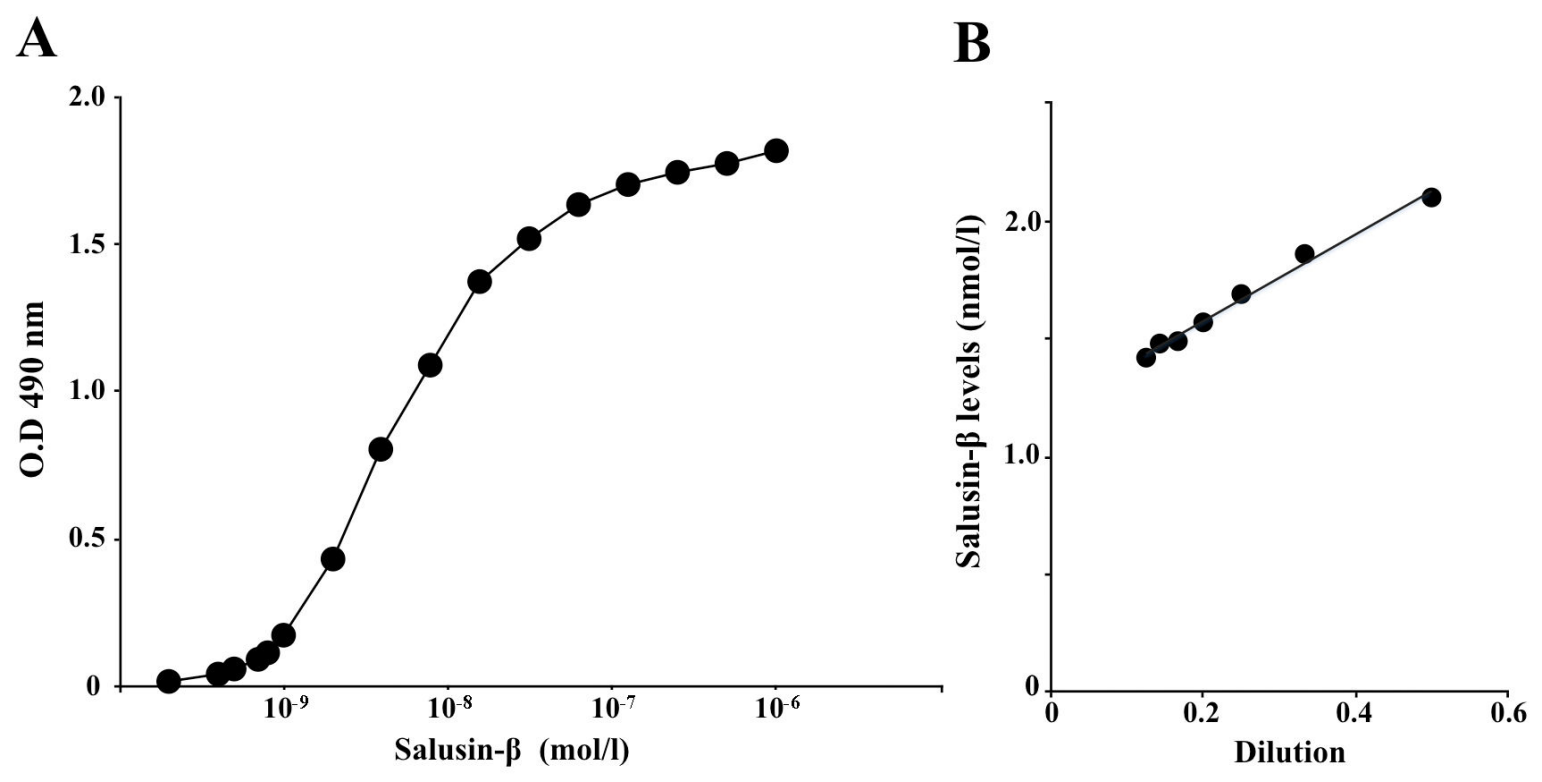
Validation of a competitive ELISA for quantifying plasma salusin-β levels in humans. **A**. Representative absorbance curve at 490 nm obtained from triplicate wells containing a range of standard human salusin-β peptides between 0.2 nmol/l and 1.0 µmol/l. **B**. Parallelism was determined by serial dilutions of pooled extracted human plasma in ELISA assay buffer to obtain 50%, 33.3%, 25%, 20%, 16.7%, 14.3%, and 12.5% plasma. Each sample was assayed in triplicate for determining salusin-β levels and linear regression was applied. Data points represent mean values obtained.

**Figure 2:**
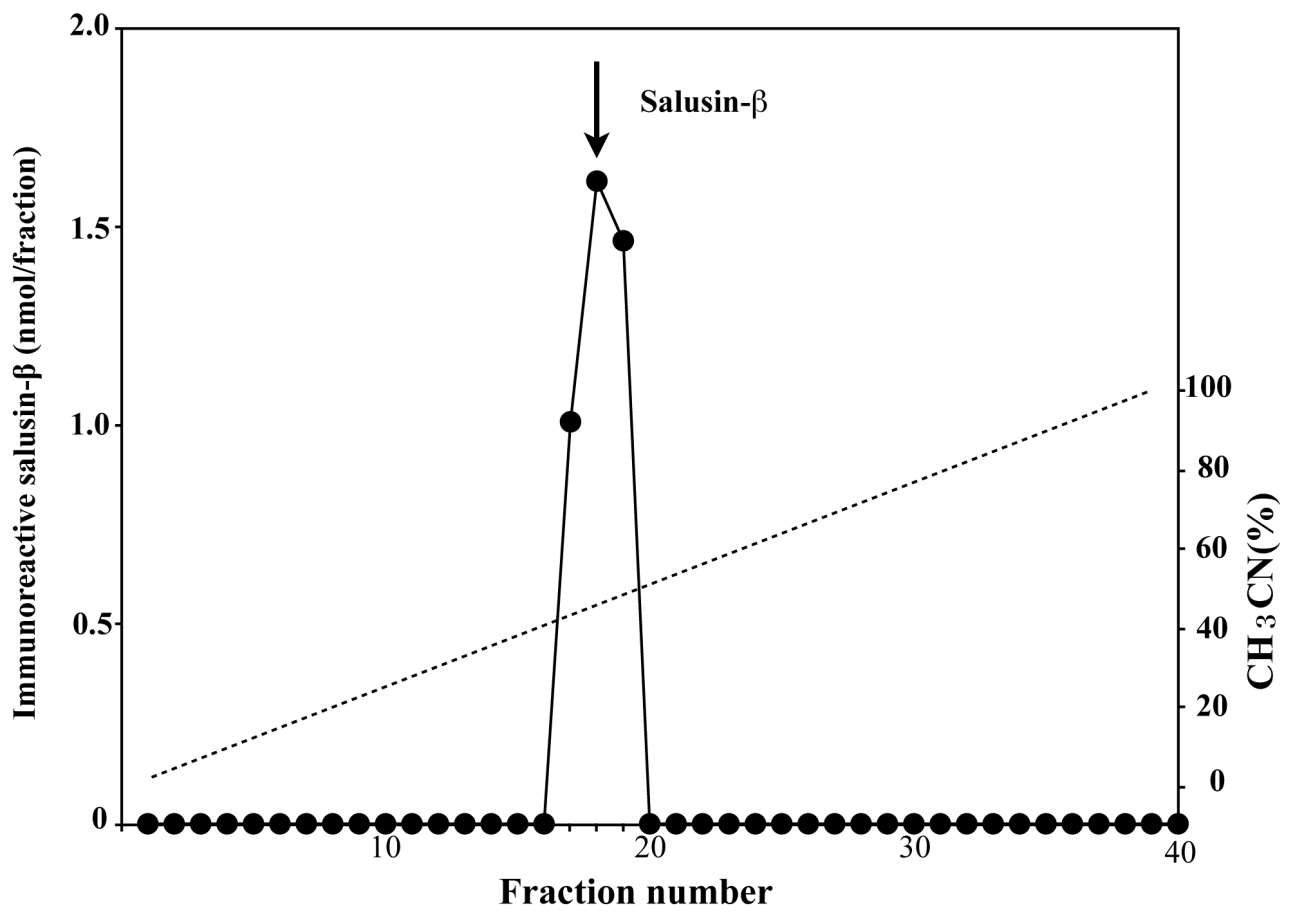
RP-HPLC analysis of salusin-β-like immunoreactivity in human plasma extract. Extracted human plasma and synthetic salusin-β were separated on an RP-HPLC column and the resultant fractions were subjected to salusin-β ELISA. Data points represent salusin-β concentrations of HPLC-fractionated extracted human plasma. The arrow indicates the fraction that contained the elution peak of synthetic salusin-β.

A standard absorbance curve was generated using a range of synthetic human salusin-β peptide diluted to the concentrations of 0.2–1000 nmol/l (Figure 1A). To evaluate parallelism, serial dilutions of extracted human plasma were assayed, and they yielded a highly linear relationship with an R^2^ value of 0.9895 (*P* < 0.0001; Figure 1B). The intra-assay CV for the extracted plasma samples was 4.5% (n = 8) and the inter-assay CV was 11.9% (n = 10). The minimum detection limit was 1.4 nmol/tube and the IC50 was 5.1 nmol/tube. Recovery from the Sep-Pak C18 column was 62.4%. The mean CV in salusin-β levels in septuplicate extractions of normal plasma samples was 6.6%. The ELISA did not cross-react with other known bioactive peptides, such as salusin-α, angiotensin I, angiotensin II, oxytocin, and arginine vasopressin. RP-HPLC fractionation of extracted human plasma and subsequent measurement of immunoreactive salusin-β showed a single peak coeluting with synthetic salusin-β peptide (Figure 2). This finding showed the successful establishment of a salusin-β ELISA suitable for detection of authentic salusin-β in human plasma.

**Figure 3:**
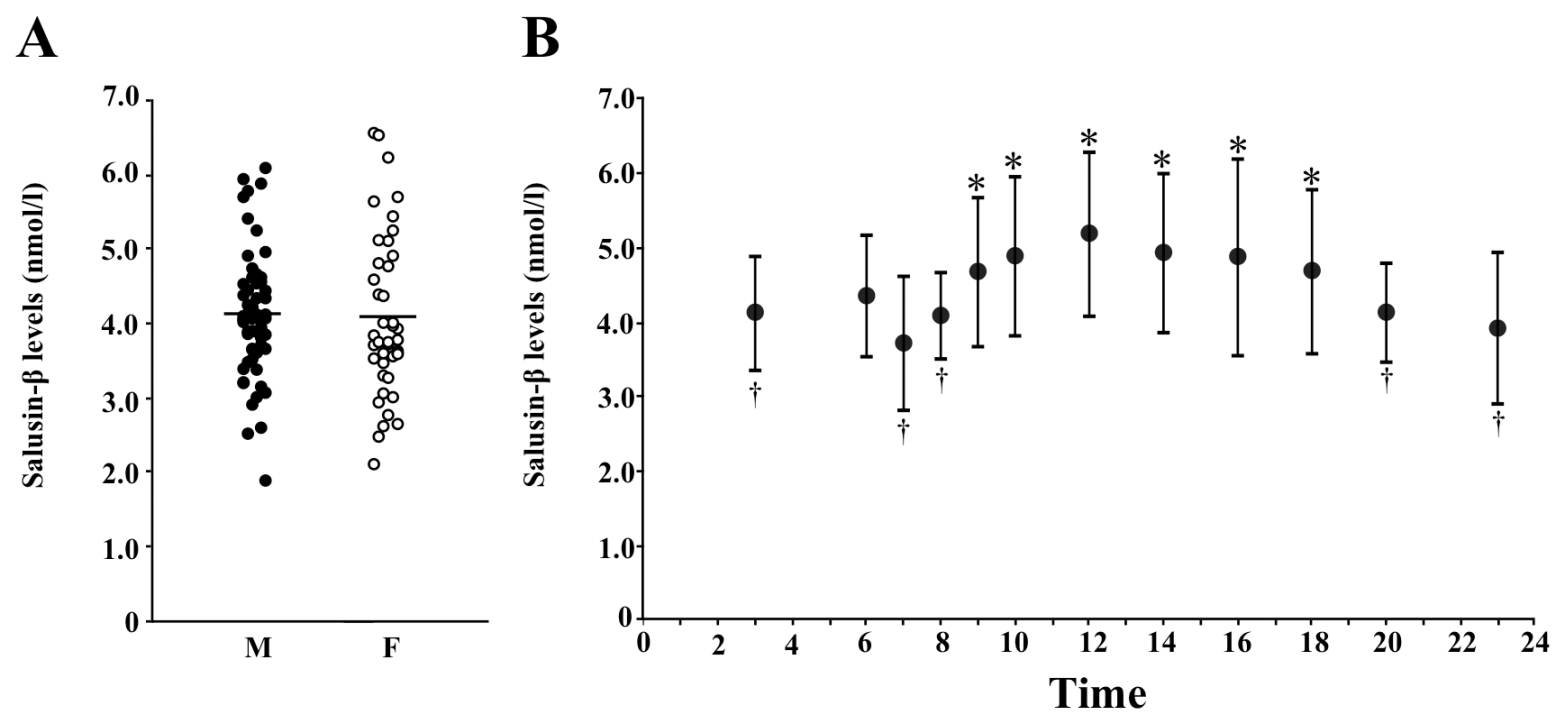
Plasma salusin-β concentrations and circadian changes in healthy volunteers. **A**. Distribution of plasma salusin-β levels obtained between 8 am and 11 am at ambulatory conditions from 106 healthy volunteers. Each point represents the salusin-β concentration found in a single subject and the horizontal bars indicate the mean salusin-β concentration for healthy males (M) and females (F). **B**. Circadian changes in mean ± SD plasma salusin-β levels in healthy subjects. *P < 0.05 vs. values at 7 am. †*P* < 0.05 vs. values at 12 noon.

The mean plasma total salusin-β concentration in 106 healthy volunteers was 4.1 ± 0.9 nmol/l, with a range of 1.9–6.6 nmol/l (Figure 3A). Repeated measurements of plasma salusin-β in healthy subjects showed a significant circadian variation. Salusin-β levels were highest during the daytime, showed a gradual decrease toward night, and reached the lowest level in the early morning (Figure 3B). Therefore, subsequent blood samplings to compare baseline values were performed between 8 am and 11 am.

**Figure 4:**
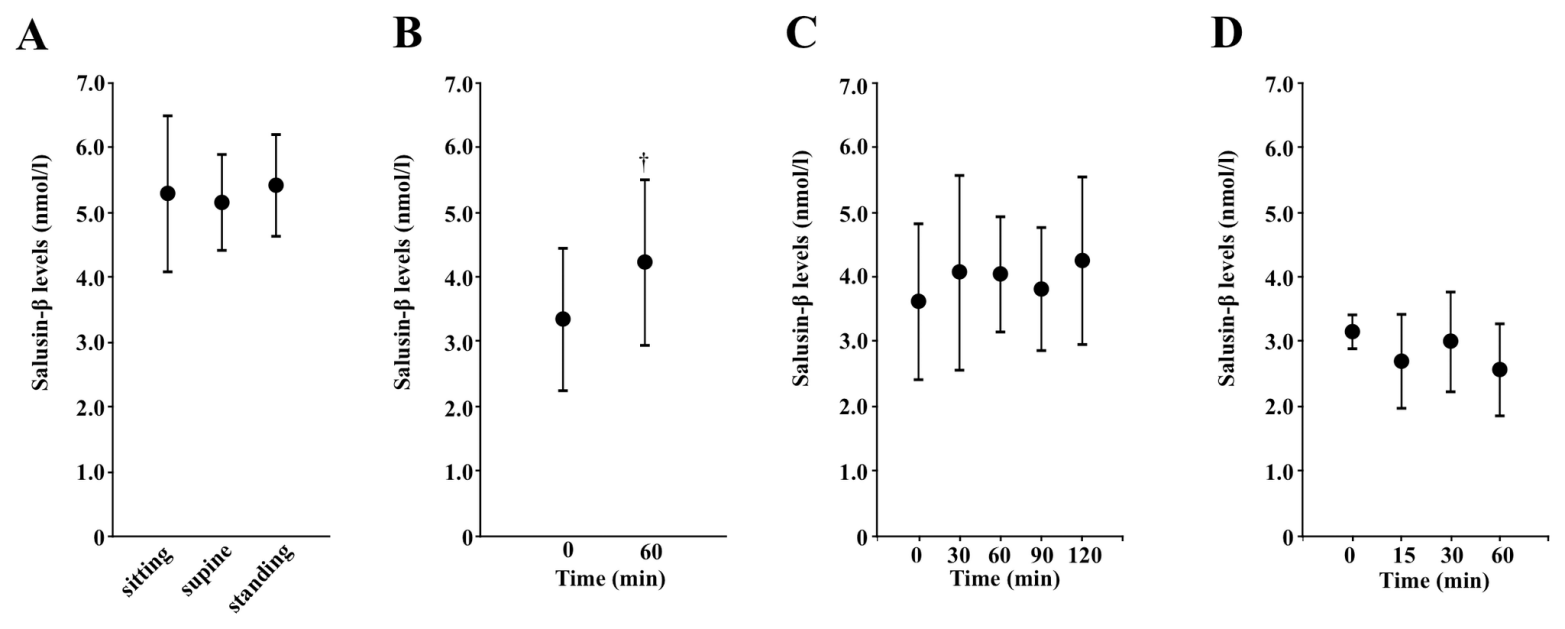
Effects of postural changes, oral water load, osmotic stimuli, and smoking on plasma salusin-β concentrations. **A**. Seven healthy subjects sat on a chair for 15 min, lay supine for the following 30 min, and then either kept standing or walking for a final 60 min. A blood sample was taken at the end of the sitting, supine, and standing periods for the measurement of plasma salusin-β levels. **B**. Nine healthy volunteers given an oral water load of 20 ml/kg body weight within 20 min had blood sampling before and 1 h after for the measurement of plasma salusin-β levels. **C**. Six healthy subjects were given hypertonic saline intravenously, and blood was drawn before and 30, 60, 90, and 120 min after the start of the infusion for the measurement of salusin-β. **D**. Six current male smokers smoked one filtered cigarette containing 0.6 mg of nicotine within 4 min. Blood samples were collected before and 15, 30, and 60 min after the initiation of smoking for the measurement of salusin-β. Data points with bars represent mean ± SD. *P < 0.05 vs. baseline value.

Because fluid restriction induces the expression of hypothalamic salusin-β in rats [3], we investigated whether plasma total salusin-β is under the influence of physiological stimuli that modulate hemodynamic and body fluid status. Plasma salusin-β levels were unaffected by 30 min in the supine position and 60-min standing position compared with the values obtained after a 15-min sitting posture (Figure 4A). Drinking 20 ml/kg body weight water did not suppress plasma salusin-β, but significantly increased its levels in 1 h (*P* < 0.05, Figure 4B).

We next examined whether physiological stimuli, which induce the release of vasopressin, could affect plasma salusin-β. Hypertonic saline infusion, a powerful stimulus of vasopressin release, did not significantly change plasma salusin-β levels (Figure 4C). Smoking also did not affect salusin-β (Figure 4D). Therefore, stimulation of vasopressin release is unaccompanied by systemic salusin-β upregulation.

**Figure 5:**
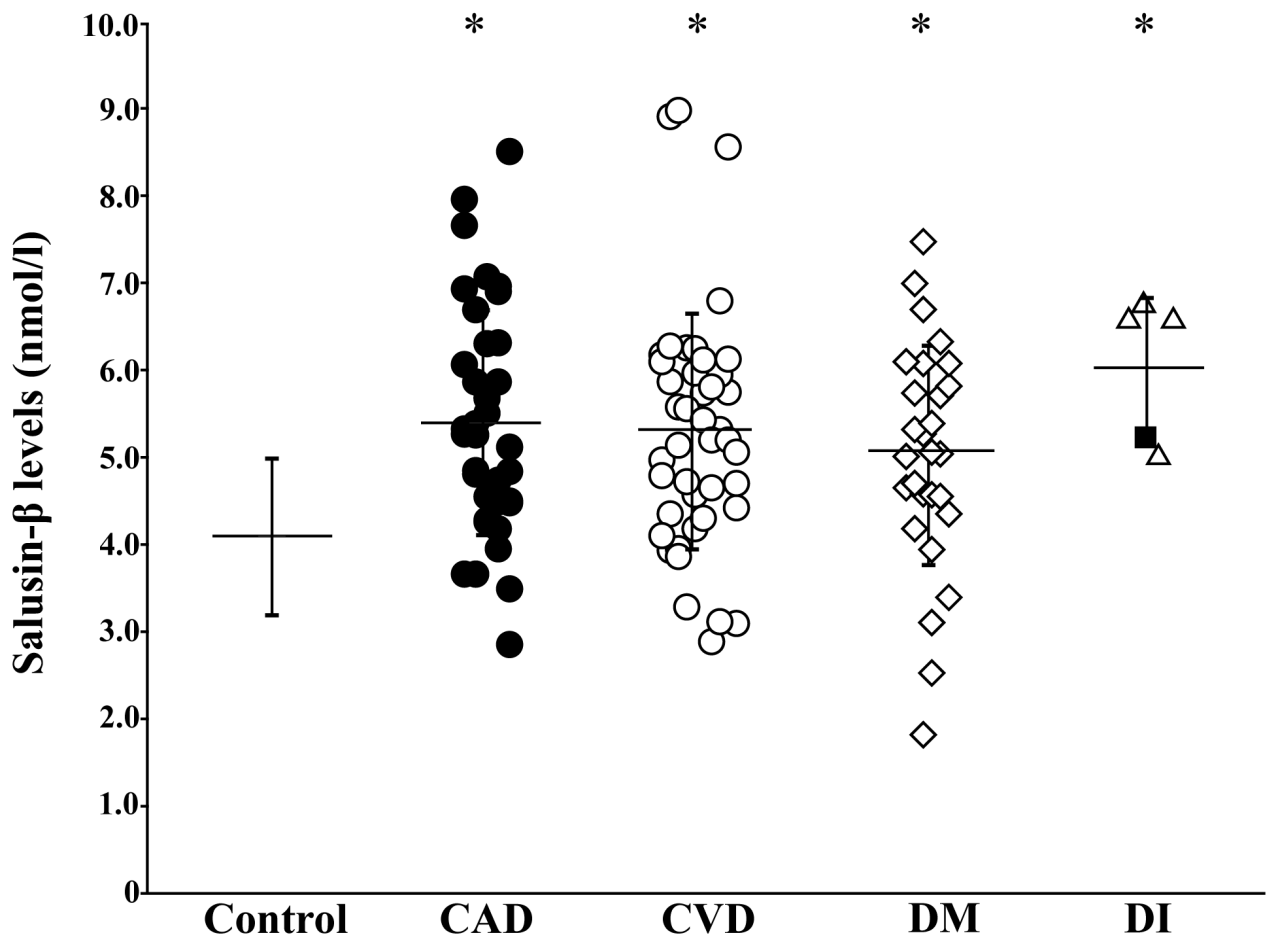
Plasma salusin-β concentrations in patients with vascular diseases, diabetes mellitus, and diabetes insipidus. Distribution of plasma salusin-β levels from 37 patients with angiographically proven coronary artery disease (CAD), 43 patients with cerebrovascular disease (CVD), 28 patients with diabetes mellitus (DM), and five patients with panhypopituitarism and complete central diabetes insipidus (DI). Each point represents the salusin-β concentration found in a single subject and the horizontal bars indicate the mean salusin-β concentration for each group. The horizontal bar with the vertical line in the left lane shows the mean ± SD of 106 healthy subjects for comparison. *P < 0.0001 vs. healthy controls. A closed square in the DI group represents the plasma salusin-β concentration of a 47-year-old man with panhypopituitarism and complete DI due to a completely severed pituitary stalk induced by a traumatic brain injury.

We measured plasma salusin-β levels in patients with cardiovascular diseases, cerebrovascular diseases, and diabetes, in addition to five patients with complete panhypopituitarism associated with central diabetes insipidus. Patients with angiographically proven coronary artery disease had significantly elevated plasma salusin-β levels compared with healthy controls (*P* < 0.0001, Figure 5). Likewise, those with cerebrovascular diseases (*P* < 0.0001) and diabetes (*P* < 0.0001) had higher plasma salusin-β levels compared with healthy controls (Figure 5). Patients with panhypopituitarism combined with complete central diabetes insipidus also had a higher plasma salusin-β levels compared with healthy controls (*P* < 0.001), which suggested that their peripheral salusin-β levels were derived from non-pituitary sources. These data demonstrate a role for plasma total salusin-β as an indicator of systemic vascular disease and diabetes, and a limited contribution of the neuroendocrine system to plasma salusin-β levels.

## Discussion

In the present study, we successfully established a sandwich ELISA suitable for the detection of immunoreactive salusin-β in human plasma. Serial dilution curves generated by extracted human plasma showed parallelism with synthetic salusin-β. Endogenous salusin-β-like immunoreactivity in normal human plasma had a single elution peak on RP-HPLC, which is identical to that of standard human salusin-β. The assay detection limit and IC50 were low, as were the intra-assay and inter-assay variations. Therefore, our ELISA specifically detects authentic salusin-β peptide circulating in human plasma.

Mean plasma salusin-β levels in our 106 healthy subjects (4.1 ± 0.9 nmol/l) are comparable with or higher than those of many other known endogenous bioactive peptides. Furthermore, these levels are two orders of magnitude higher than serum salusin-α [15-17], which is derived from a common precursor, preprosalusin. This discrepancy could be due to differential expression and distribution of salusin-α and salusin-β proteins at each organ or tissue [2,4]. Alternatively, this discrepancy could be because the current assay uses column extraction of plasma, which theoretically releases and detects salusin-β molecules bound to serum proteins. At present, we are unable to directly measure the concentrations of free salusin-β peptides because unextracted plasma contains proteins interfering with the ELISA assay. In rats, significant reductions in blood pressure and heart rate were induced by intravenous administration of 0.1–1 ng/kg salusin-β, which is expected to raise the peak serum level to as high as 1–10 pmol/l [5,6]. Estimated effective concentrations of free salusin-β, which acutely modulate systemic hemodynamics, are lower than the total plasma salusin-β levels as demonstrated in the present study. Circulating salusin-β may be mostly bound to plasma proteins, and thus are devoid of potent hemodynamic activity. Although our data are still in agreement with the hypothesis that circulating free salusin-β may function as cardiotropic peptide hormone that modulates hemodynamics in humans [18,19], further studies are required to separate free and bound salusin-β peptide circulating in human plasma.

Neuroendocrine salusin-β is localized to vasopressin-containing neurons and stimulates the release of arginine vasopressin and oxytocin in an autocrine/paracrine fashion [1-4]. Chronic osmotic stimuli, such as salt loading and dehydration, induce the expression of salusin-β in the hypothalamo-neurohypophyseal system, which in turn, stimulates vasopressin and oxytocin release from nerve endings [3]. Upregulated salusin-β in the supraoptic nucleus and the paraventricular nucleus can be transported to the internal zone of the median eminence and the neurohypophysis, and secreted from nerve terminals in the neurohypophysis [1,3]. However, whether circulating salusin-β in human plasma is mainly derived from the pituitary and whether vasopressin release is associated with increased peripheral salusin-β levels, which could in turn affect systemic hemodynamics and atherosclerosis, remain undetermined. The present study demonstrated that plasma salusin-β concentrations fluctuate differently from many other fluid-regulating molecules, such as vasopressin and aldosterone. Our results further indicated that plasma total salusin-β levels were not affected by potent vasopressin stimuli, such as hypertonic saline infusion or smoking, or by postural changes. Furthermore, our five patients with panhypopituitarism combined with complete diabetes insipidus showed higher plasma salusin-β level than normal values. These data indicate the limited contribution of neuroendocrine sources to peripheral total salusin-β levels in humans.

The present study also showed a significant circadian variation of plasma salusin-β with an early morning nadir. This diurnal change appears to be more dependent upon sleep rather than chronological time. This is because subjects who showed the lowest levels at approximately 7 am and a subsequent rise in plasma salusin-β levels after 9 am when they got up at 6 am, later showed a delayed nadir and subsequent rise when they fell asleep again after blood withdrawal at 7 am and getting out of bed later (data not shown). The mechanism behind this early morning decrease is unknown. However, because the major origin of total plasma salusin-β levels is not the neuroendocrine system, it should be under the control of unidentified systemic tissues/cells producing and secreting salusin-β peptide.

Another remarkable finding of the current study is that plasma salusin-β levels were significantly increased in patients with coronary artery disease, cerebrovascular disease, and diabetes compared with healthy volunteers. Salusin-β is a potent endogenous atherogenic factor produced and secreted by infiltrating macrophages, and it acts on these macrophages in an autocrine/paracrine fashion to induce foam cell formation [7,8]. Locally produced salusin-β may also act on adjacent endothelial cells to accelerate inflammatory responses [20]. Therefore, elevated peripheral salusin-β levels could represent the amount of peptide secreted by macrophages in response to vascular inflammatory processes. Recent studies have reported that serum salusin-α levels, which possess contrasting antiatherosclerotic activity by suppressing macrophage foam cell formation, are lower in patients with coronary artery disease, hypertension, and renal failure than in healthy controls, and the magnitude of decrease in serum salusin-α appears pronounced as atherosclerosis advances [7,15,17,21]. Therefore, salusin-α has been proposed as a candidate biomarker for atherosclerotic and cardiovascular diseases in humans [18,19]. Our present study showing higher salusin-β levels in patients with definite evidence of coronary artery disease, cerebrovascular disease, and diabetes, suggests the possibility that plasma salusin-β could also serve as an indicator for an enhanced systemic atherogenic process.

In summary, we successfully established a sensitive and specific ELISA for detecting total human plasma salusin-β levels, which are considered to be the summation of circulating free and bound salusin-β peptide. Salusin-β levels are sufficient to modulate hemodynamics, but are not under regulation of osmotic stimuli or postural changes.
