# Correction: Circulating Levels of Human Salusin-β, a Potent Hemodynamic and Atherogenesis Regulator

**DOI:** 10.1371/annotation/f628c1d6-4bee-4725-b434-690f169a7849

**Published:** 2013-11-15

**Authors:** Kazumi Fujimoto, Akinori Hayashi, Yuji Kamata, Akifumi Ogawa, Takuya Watanabe, Raishi Ichikawa, Yoshitaka Iso, Shinji Koba, Youichi Kobayashi, Takatoshi Koyama, Masayoshi Shichiri

There were multiple errors in the title. The correct title is: "Circulating Levels of Human Salusin-β, a Potent Hemodynamic and Atherogenesis Regulator." The correct citation is: Fujimoto K, Hayashi A, Kamata Y, Ogawa A, Watanabe T, et al. (2013) Circulating Levels of Human Salusin-β, a Potent Hemodynamic and Atherogenesis Regulator. PLoS ONE 8(10): e76714. doi:10.1371/journal.pone.0076714. 

